# Improving Phrase Segmentation in Symbolic Folk Music: A Hybrid Model with Local Context and Global Structure Awareness

**DOI:** 10.3390/e27050460

**Published:** 2025-04-24

**Authors:** Xin Guan, Zhilin Dong, Hui Liu, Qiang Li

**Affiliations:** 1 School of Microelectronics, Tianjin University, Tianjin 300072, China; guanxin@tju.edu.cn (X.G.); lin_lin01@tju.edu.cn (Z.D.); 2 School of Music and Film, Tianjin Normal University, Tianjin 300387, China

**Keywords:** music phrase segmentation, symbolic music, attention mechanism

## Abstract

The segmentation of symbolic music phrases is crucial for music information retrieval and structural analysis. However, existing BiLSTM-CRF methods mainly rely on local semantics, making it difficult to capture long-range dependencies, leading to inaccurate phrase boundary recognition across measures or themes. Traditional Transformer models use static embeddings, limiting their adaptability to different musical styles, structures, and melodic evolutions. Moreover, multi-head self-attention struggles with local context modeling, causing the loss of short-term information (e.g., pitch variation, melodic integrity, and rhythm stability), which may result in over-segmentation or merging errors. To address these issues, we propose a segmentation method integrating local context enhancement and global structure awareness. This method overcomes traditional models’ limitations in long-range dependency modeling, improves phrase boundary recognition, and adapts to diverse musical styles and melodies. Specifically, dynamic note embeddings enhance contextual awareness across segments, while an improved attention mechanism strengthens both global semantics and local context modeling. Combining these strategies ensures reasonable phrase boundaries and prevents unnecessary segmentation or merging. The experimental results show that our method outperforms the state-of-the-art methods for symbolic music phrase segmentation, with phrase boundaries better aligned to musical structures.

## 1. Introduction

Musical phrases serve as fundamental semantic units in musical compositions, analogous to sentences in natural language [1]. Each phrase typically forms a structurally complete unit, with closely interwoven internal elements that create a cohesive whole [2]. In musical performance, phrases function as core units for expressing emotions, conveying information, and presenting themes, making precise segmentation essential for understanding melodic emotional expression and information transmission [3]. However, unlike natural language, which utilizes punctuation as explicit segmentation markers, musical phrases lack such clear indicators, necessitating specialized analytical methods to determine phrase boundaries [4].

Initially, researchers adopted rule-based methods for phrase segmentation, relying on explicit segmentation cues such as long notes and rests, alongside handcrafted rules and fixed weights [5]. However, this approach significantly limited the adaptability of models to various musical styles [6]. In contrast, traditional probabilistic machine learning methods addressed some of these limitations by dynamically adjusting feature weights using probabilistic models [7]. Nevertheless, these methods still required manually designed feature templates, which often failed to fully capture the diversity of musical contexts, leading to less effective segmentation across varying compositions.

With the rapid development of deep learning, particularly advancements in natural language processing (NLP), symbolic music segmentation has taken new directions [8]. Analogous to sequence labeling tasks in NLP, symbolic music segmentation involves labeling each “token” (in this case, each note). Building on this similarity, Zhang et al. [9] applied the widely used BiLSTM-CRF model from sequence labeling tasks to symbolic music segmentation, achieving impressive results. However, unlike typical sequence labeling tasks, symbolic music segmentation requires not only local contextual information but also global context to effectively identify phrase boundaries [10]. Tillmann’s study highlights that global context not only filters out local noise but also reveals long-term structural features of music, enabling more precise structural analysis [11]. Consequently, integrating both global and local contextual information has become a central focus in advancing symbolic music phrase segmentation.

The advent of Transformer models [12] has further expanded the potential for incorporating global context into symbolic music segmentation. With their powerful multi-head self-attention mechanism, Transformer models excel at capturing global dependencies. However, directly applying Transformers to musical phrase segmentation poses challenges in learning local contextual information. These challenges stem from structural differences: Transformers use a parallel architecture, while BiLSTMs rely on a sequential structure that inherently captures local dependencies within phrases [13]. Although Transformers employ positional encoding and self-attention mechanisms to learn context, they often emphasize global dependencies at the expense of local contextual nuances essential for intra-phrase dependency learning [14].

Additionally, symbolic music differs significantly from natural language in its processing requirements. Unlike natural language, where punctuation clearly delineates syntactic structures, symbolic music lacks explicit segmentation markers, complicating the modeling of hierarchical musical structures [15]. Traditional multi-head self-attention mechanisms, without guided learning for local phrase structures, struggle to effectively capture local contextual information.

Moreover, in traditional Transformer models, the embedding layer relies on static word vectors, similar to the polysemy issue in natural language processing [16]. The semantic information of each note is highly context-dependent. In symbolic music, the meaning of a note often depends on its contextual information, particularly its position within the melody and the rhythm pattern. However, this static embedding approach lacks dynamic adjustment to contextual information [17], which limits the model’s flexibility in adapting the representation of notes. This becomes particularly problematic when the same note appears in different rhythmic patterns, as the model struggles to understand the local contextual information, failing to accurately identify phrase boundaries based on the specific context, thus impacting the precision of phrase segmentation.

To address these challenges, this paper proposes an improved model that integrates both local context and global structure awareness. First, to strengthen the model’s ability to learn overall musical structure, we incorporate a Transformer model. Second, to enhance the learning of local contextual information, we introduce intra-phrase self-attention heads that guide local context modeling. Additionally, by replacing traditional static word embeddings with Embeddings from Language Models (ELMos), we improve the model’s sensitivity to local context. Finally, by combining these two approaches, we ensure the rationality of phrase boundaries, effectively preventing over-segmentation or merging errors.

## 2. Related Work

Tenney and Polansky [5] were among the first to propose rule-based methods for symbolic music phrase segmentation. Their approach identified phrase boundaries by applying explicit rules, such as the presence of long notes and rests. Building on this foundation, Frankland [18] applied the Generative Theory of Tonal Music (GTTM), originally proposed by Lerdahl and Jackendoff, to phrase segmentation from a music-theoretical perspective. GTTM replaced traditional rule-based analysis with grouping and preference rules, offering a systematic and hierarchical framework for segmentation. Eugene Narmour’s Implication-Realization (IR) theory [19], in contrast, applied principles of perceptual psychology, emphasizing the relational dynamics between notes in the segmentation process.

Among rule-based methods, the local boundary detection model (LBDM) [20] has gained widespread adoption. This model segments phrases by defining rules to detect significant changes in pitch or duration. While rule-based methods have provided robust frameworks for phrase segmentation, they rely heavily on expert-designed rules and fixed weights. This dependence introduces several limitations: fixed rules and weights lack the flexibility to adapt to various musical styles, and the manual design and adjustment process is labor-intensive, making it difficult to accommodate complex and diverse musical structures. As a result, these methods face significant challenges in scalability and generalization.

With advancements in machine learning, probabilistic methods have emerged as an alternative for music phrase segmentation. These methods overcome the rigidity of fixed rule weights by learning relationships between features from data, thereby improving model adaptability. Unlike rule-based methods, probabilistic approaches leverage predefined feature templates to dynamically learn weight distributions for phrase segmentation. Notable examples include Maximum Entropy Models (MaxEnts) [21] and Restricted Boltzmann Machines (RBMs) [22]. In subsequent research, Michel et al. [23] proposed a Hidden Markov Model (HMM)-based method, which models musical sequences and uses prediction errors between expected and actual notes to identify phrase boundaries, reframing boundary recognition as a sequence prediction problem. Shahaf Bassan [24] further introduced the Time Prediction Error Integration method, which employs a time prediction model to detect significant deviations in features, identifying phrase boundaries in regions with large prediction errors.

Although probabilistic methods address the inflexibility of fixed weights in rule-based approaches, they encounter two primary challenges: (1) they rely heavily on manually designed feature templates, which often fail to capture the diversity and complexity of musical structures, and (2) they lack the capability to dynamically adapt feature extraction to different musical styles and contexts, limiting their effectiveness in capturing rich contextual information.

With the advent of deep learning, neural network-based methods have gained considerable attention in music phrase segmentation tasks. Building on traditional machine learning approaches, researchers have increasingly explored deep learning models for sequence modeling in music. Michel et al. [23] applied Recurrent Neural Networks (RNNs) to music sequence modeling, demonstrating significant improvements in phrase segmentation accuracy over Hidden Markov Models (HMMs) by leveraging prediction errors. Wang et al. [25] employed Long Short-Term Memory (LSTM) networks, utilizing the norm of hidden outputs at each time step to identify phrase boundaries. Inspired by the widespread application of BiLSTM-CRF models in sequence labeling tasks, Zhang et al. [9] applied BiLSTM-CRF to symbolic music phrase segmentation, achieving state-of-the-art performance. However, while the BiLSTM-CRF model excels at capturing contextual information, its outputs rely heavily on local contextual dependencies, offering limited consideration of global context. This constraint reduces the model’s effectiveness in handling phrases that require a comprehensive understanding of global contextual information.

To address these challenges, this paper introduces a phrase segmentation method that fully integrates local context and global structure awareness. The proposed approach captures and synthesizes multi-level contextual information in music, equipping the model with enhanced comprehension capabilities. By combining local context and global structure, the model effectively identifies short-term dependencies between notes while simultaneously capturing the overarching structural features of musical compositions. This dual integration significantly improves segmentation accuracy and enhances adaptability to diverse musical styles.

## 3. Method

### 3.1. Problem Definition

Let $X=\{X_{1},X_{2},\ldots ,X_{T}\}$ denote a symbolic music sequence of *T* notes, where each $X_{i}$ represents a note in a melodic sequence. The task is to identify the phrase boundaries within this sequence by determining whether each note marks the end of a musical phrase.

For each note *i*, we define a binary label $y_{i}$ to indicate whether it is the final note of a phrase:

(1) $$y_{i}=\left\{\begin{array}{cc} 1, & \mathrm{if}\;\mathrm{note}\;i\;\mathrm{is}\;\mathrm{the}\;\mathrm{last}\;\mathrm{note}\;\mathrm{of}\;a\;\mathrm{phrase}\\ 0, & \mathrm{otherwise} \end{array}\right.$$

Let $Y=\{y_{1},y_{2},\ldots ,y_{T}\}$ denote the sequence of binary labels corresponding to *X*, where $y_{i}\in \left\{0,1\right\}$ indicates whether the *i*-th note is a phrase boundary.

The objective is to predict the label ${\hat{y}}_{i}$ for each note i∈{1,2,…,T}, learning to determine whether each note is a phrase boundary.

### 3.2. Model Architecture

**Figure 1:**
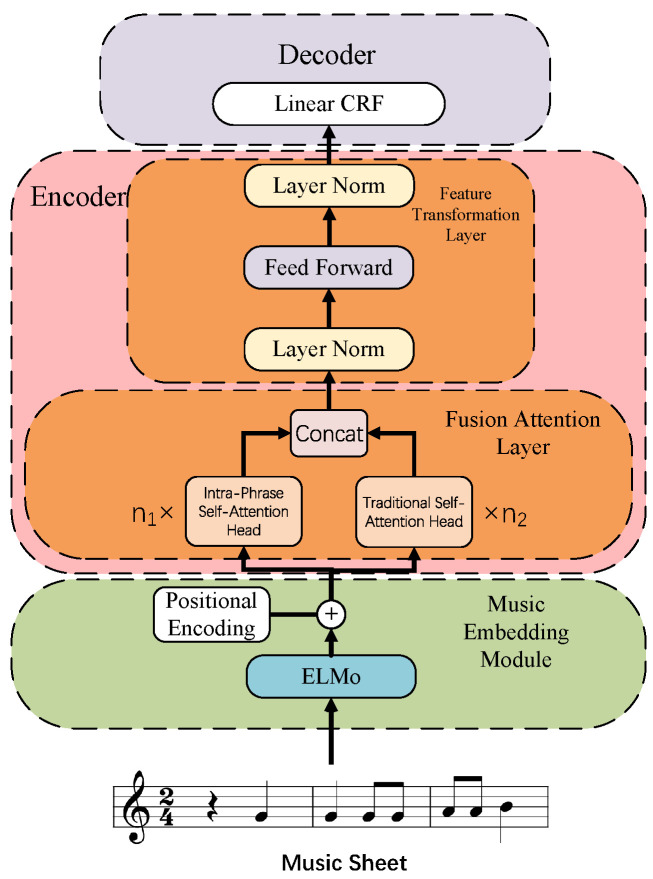
Block diagram of model structure.

Our model architecture comprises a music embedding module, an encoder, and a decoder, as shown in Figure 1. The music embedding module incorporates an ELMo embedding model and a positional encoder. The encoder consists of a single-layer Transformer encoder without residual connections, integrated with a Fusion Attention Layer. The decoder is implemented using a linear Conditional Random Field (CRF) layer. The details are as follows:

### 3.3. Music Embedding Module

This module is designed to transform each note vector from the original musical score into a word embedding vector that integrates both contextual information and positional encoding.

Initially, the module transforms the musical score into a list of vectors, where each token is encoded into a vector representation with five dimensions:

(2) $$\;X_{t}=\left\{{\mathrm{bar}}_{t},{\mathrm{position}}_{t},{\mathrm{pitch}}_{t},{\mathrm{duration}}_{t},\mathrm{time}\;{\mathrm{signature}}_{t}\right\},\;t=1,\;2,\ldots ,n$$

Let the input sequence of tokens be

(3) $$X=\{X_{1},X_{2},\ldots ,X_{n}\}$$

Next, the note vectors are processed through the ELMo model to compute the contextual embedding for each time step:

(4) $$T_{t}=\mathrm{ELMo}\left(X_{t}\right)=\gamma \sum_{l=1}^{L}s_{l}H_{l,t}$$

where the notations are defined as follows:

- $T_{t}$ represents the contextual embedding at the *t*-th time step.
- $\mathrm{ELMo}(X_{t})$ is the embedding computed by the ELMo model at the *t*-th time step.
- γ is a trainable scalar parameter that adjusts the dynamic range of the contextual embedding.
- *L* is the number of bidirectional LSTM layers (set to 2 in this case).
- $s_{l}$ is the weight for the *l*-th layer, defined as
  (5) $$s_{l}=\frac{\exp(w_{l})}{\sum_{k=1}^{L}\exp\left(w_{k}\right)}$$
  where $w_{l}$ is a learnable parameter for the *l*-th layer.
- $H_{l,t}$ represents the output of the *l*-th bidirectional LSTM layer at time step *t*, which incorporates contextual information from both the forward and backward LSTMs:
  (6) $$H_{l,t}=\left[H_{l,t}^{\mathrm{forward}};H_{l,t}^{\mathrm{backward}}\right]$$

Since the embedded word vectors lack positional information, sequence information for the notes is incorporated using conditional positional encoding (*P*), defined as

(7) $$P\left(t,2i\right)=\sin\left(\frac{t}{10000^{2i/d}}\right)$$

(8) $$P\left(t,2i+1\right)=\cos\left(\frac{t}{10000^{2i/d}}\right)$$

where the notations are defined as follows:

- *t* is the time step index.
- *i* is the dimension index.
- *d* is the embedding dimension.

Finally, the input matrix to the encoder is computed as

(9) $$H^{\prime }=T+P$$

This formulation ensures that the input representation includes both contextual embeddings and positional information for effective sequence modeling.

### 3.4. Encoder

The encoder module is designed to extract musical context information from the score data processed by the word embedding module and integrate this information into each time step. The specific process is as follows.

#### 3.4.1. Fusion Attention Layer

The music context extraction module is designed to capture both local context and global structure from the musical score. This module employs a multi-head self-attention mechanism, with specific heads dedicated to extracting either local context. The detailed process is as follows.

Computing the Attention-Based Contextual Representation:

For the input $H^{\prime }$, which incorporates positional encoding, we first apply linear transformations to compute the query (*Q*), key (*K*), and value (*V*) matrices for each attention head. For a specific attention head *i*, the transformations are as follows:

(10) $$Q_{i}=H^{\prime }\cdot W_{Q_{i}},\;K_{i}=H^{\prime }\cdot W_{K_{i}},\;V_{i}=H^{\prime }\cdot W_{V_{i}}$$

where $W_{Q_{i}},W_{K_{i}},$ and $W_{V_{i}}\in R^{H\times H}$ are the learnable weight matrices for the *i*-th attention head, and $H^{\prime }$ is the output of the ELMo model combined with positional encoding.

Next, the self-attention mechanism calculates the similarity between the query ($Q_{i}$) and key ($K_{i}$) matrices to derive the attention weight matrix for the *i*-th head. This is achieved by computing the dot product of $Q_{i}$ and $K_{i}$, scaling the result to prevent instability from large values, and normalizing it using the softmax function:

(11) $$A_{i}=\mathrm{softmax}\left(\frac{Q_{i}\cdot K_{i}^{⊤}}{\sqrt{H}}\right)$$

Here, the following are denoted:

- $A_{i}\in R^{T\times T}$: The attention weight matrix for the *i*-th head, representing the dependencies between each position in the sequence.
- $\sqrt{H}$: A scaling factor, typically set to the square root of the hidden size, to stabilize gradients during training.

Using the attention weight matrix $A_{i}$, the value matrix $V_{i}$ is weighted and summed to compute the contextual representation for the *i*-th head:

(12) $$O_{i}=A_{i}\cdot V_{i}$$

The output *O* represents the weighted contextual embedding for each note within the sequence under the self-attention mechanism. Each note’s representation in *O* is enriched with information from its surrounding context, effectively capturing both local and global dependencies within the sequence. This enhanced representation forms the foundation for subsequent processing in the model.

Loss-Constrained Local Self-Attention Value Calculation:

To ensure that the local self-attention heads focus on capturing contextual information within a phrase rather than the global context of the entire musical score, we introduce a target attention distribution specific to intra-phrase relationships and a custom loss function to guide the learning process.

The goal is to minimize the difference between the attention distribution *A* computed by the model and the target attention distribution $A_{\mathrm{target}}$. This difference is quantified using the Kullback–Leibler (KL) divergence loss:

(13) $$L_{Attention}=\sum_{i=1}^{T}\sum_{j=1}^{T}A_{\mathrm{target}}\left[i,j\right]\cdot \log\left(\frac{A_{\mathrm{target}}\left[i,j\right]}{A[i,j]}\right)$$

where the notations are defined as follows:

- $A_{\mathrm{target}}\left[i,j\right]$: The target attention distribution.
- A[i,j]: The attention distribution computed by the self-attention mechanism.
- *T*: The length of the input sequence.

Target Attention Distribution:

The target attention distribution $A_{\mathrm{target}}$ is defined as

(14) $$A_{\mathrm{target}}\left[i,j\right]=\left\{\begin{array}{cc} \frac{1}{|s_{k}|}, & \mathrm{if}\;i,j\in \mathrm{same}\;\mathrm{phrase}\;s_{k}\\ 0, & \mathrm{if}\;i,j\in \mathrm{different}\;\mathrm{phrases} \end{array}\right.$$

where the notations are defined as follows:

- $s_{k}$: The *k*-th phrase in the sequence.
- $|s_{k}|$: The length of phrase $s_{k}$.

- If *i* and *j* belong to the same phrase $s_{k}$, the attention value $A_{\mathrm{target}}\left[i,j\right]$ is uniformly distributed within the phrase as $\frac{1}{|s_{k}|}$.
- If *i* and *j* belong to different phrases, the attention value $A_{\mathrm{target}}\left[i,j\right]$ is set to 0, effectively eliminating cross-phrase dependencies.

The KL divergence loss optimizes the model by aligning the computed attention distribution *A* with the target distribution $A_{\mathrm{target}}$. This ensures that the attention mechanism prioritizes intra-phrase contextual dependencies, enhancing its ability to capture meaningful relationships within phrases.

Concatenating Multi-Head Attention Outputs:

Finally, the outputs from the self-attention heads—designed to capture both local and global contextual information—are concatenated to form the final output. This step combines the diverse contextual representations learned by each attention head into a unified representation:

(15) $$O=\mathrm{Concat}(O_{1},O_{2},\ldots ,O_{n_{1}+n_{2}})$$

Here, the concatenation operation ensures that information from all attention heads is integrated, providing a comprehensive representation for further processing.

#### 3.4.2. Feature Transformation Layer

This layer is designed to further process and transform the output from the Fusion Attention Layer before passing it to the decoder. Specifically, it normalizes the output of the Fusion Attention Layer, applies a feedforward network, and concludes with another layer normalization to produce the final encoder output. The detailed computation is as follows:

(16) $$X^{\prime }=\mathrm{Norm}\left(\mathrm{ReLU}\left(\mathrm{Norm}\left(OW_{1}+b_{1}\right)W_{2}+b_{2}\right)\right)$$

where the notations are defined as follows:

- *O*: The unnormalized input (output of the self-attention layer).
- $W_{1},W_{2}$: Weight matrices for the first and second linear layers, respectively.
- $b_{1},b_{2}$: Bias terms for the first and second linear layers, respectively.

Steps:

1. The input *O* is processed through a linear transformation:
  (17) $$OW_{1}+b_{1}$$
  followed by the ReLU activation function.
2. The output from the ReLU activation ($F_{1}$) is passed through a second linear transformation:
  (18) $$F_{1}W_{2}+b_{2}$$
3. Finally, the entire output $X^{\prime }$ undergoes a second normalization:
  (19) $$X^{\prime }=\mathrm{Norm}\left(X^{\prime }\right)$$
  ensuring stability during training by standardizing the features.

This sequence of transformations refines the extracted features, ensuring that they are well suited for decoding.

### 3.5. Decoder

The decoder module is responsible for decoding the transformed feature output into the corresponding label sequence. Specifically, this module consists of a linear layer followed by a Conditional Random Field (CRF) layer. The detailed computation process is as follows:

Each $X_{t}^{\prime }$ is passed through a fully connected linear layer to project the features into a label space. This operation is represented mathematically as

(20) $$z_{t}=W_{3}X_{t}^{\prime }+b$$

The output ${\hat{y}}_{t}$ (predicted value) is further processed to produce probabilities for each possible label (1 or 0). This is achieved using the sigmoid activation function:

(21) $$p\left({\hat{y}}_{t}=1|X_{t}^{\prime }\right)=\frac{1}{1+e^{-z_{t}}}$$

Finally, the CRF layer models the dependencies between adjacent labels using a transition matrix *T*, which assigns scores for transitioning between different states. The probability of a sequence of labels $y=\{y_{1},y_{2},\ldots ,y_{n}\}$ is given by

(22) $$P\left(y|z\right)=\frac{\exp\left(\sum_{t=1}^{n}T_{y_{t-1},y_{t}}+z_{t}\right)}{\sum_{y^{\prime }}\exp\left(\sum_{t=1}^{n}T_{y_{t-1}^{\prime },y_{t}^{\prime }}+z_{t^{\prime }}\right)}$$

During inference, the CRF layer uses the Viterbi algorithm to find the most likely sequence of labels ${\hat{y}}^{*}$, which maximizes the sequence probability [26]:

(23) $${\hat{y}}^{*}=\arg\max_{y}P\left(y|z\right)$$

### 3.6. The Loss Function

#### 3.6.1. Sequence Labeling Loss

The sequence labeling loss $L_{\mathrm{Labeling}}$ is computed using binary cross-entropy loss for each time step in the sequence. The formula is as follows:

(24) $$L_{\mathrm{Labeling}}=-\frac{1}{n}\sum_{t=1}^{T}\left[y_{t}\log{\hat{y}}_{t}+\left(1-y_{t}\right)\log\left(1-{\hat{y}}_{t}\right)\right]$$

where the notations are defined as follows:

- $y_{t}$ is the true label at time step *t*.
- ${\hat{y}}_{t}$ is the predicted value at time step *t*.
- *T* is the length of the sequence.
- *n* is the total number of sequences.

#### 3.6.2. Total Loss

The total loss function combines the target attention loss and the sequence labeling loss as follows:

(25) $$L=\alpha L_{Labeling}+\beta L_{Attention}$$

where the notations are defined as follows:

- $L_{Labeling}$ is the sequence labeling loss.
- $L_{Attention}$ is the KL divergence loss for the target attention.
- α and β are hyperparameters that control the weights of the two losses in the total loss function.

## 4. Experiment and Results

In this section, we first introduce the dataset in Section 4.1, followed by the training details in Section 4.2. Section 4.3 presents a comparison between our model and other models. In Section 4.4, we conduct ablation experiments to evaluate the impact of the model components on its performance. In Section 4.5, we perform attention heatmap visualization. Finally, in Section 4.6, we discuss and summarize our experimental results.

### 4.1. Dataset

The dataset used in this study is the Essen Folksong Collection (EFSC), compiled with data starting from 1982 by Helmuth Schaffrath and his team at the Essen Music Academy in Germany [27]. EFSC converts printed music into a computer-readable format using the “Essen Associative Code” (EsAC), and currently contains 6236 extensively annotated folk songs. Each folk song is accompanied by annotations in EsAC format, which meticulously include pitch, time signature, and phrase boundaries marked by music experts. Due to its rich and accurate annotations, EFSC has become a vital resource for training and testing phrase segmentation models and is widely considered a benchmark dataset for evaluating the accuracy of phrase segmentation [9].

### 4.2. Data Preparation and Model Training

During model training, 90% of the data were used for the training set, with the remaining 10% randomly allocated as the test set. Additionally, the training set underwent data augmentation. Specifically, the pitch of each song was transposed within an octave range (from −12 to +12 semitones) to help the model not only learn the relative relationships within phrases but also remain robust to absolute pitch changes. This process enhanced the model’s understanding of musical contexts.

#### Experimental Setup

This study uses the PyTorch (version 1.13.1) deep learning framework and was trained on hardware configured with an Intel i7-14700K CPU (Intel Corporation, Santa Clara, CA, USA) and an NVIDIA 4070 Ti GPU (NVIDIA Corporation, Santa Clara, CA, USA). The model setup is as follows: the Adam optimizer was used, and a Dropout layer was incorporated to prevent overfitting. During training, we employed 5-fold cross-validation and an early stopping strategy. After each training epoch, the model’s performance on the validation set was evaluated, and the learning rate was automatically adjusted based on the validation loss to ensure the model’s accuracy and reliability. The specific settings are shown below.

Hyperparameter Settings:

- ELMo Embedding Dimension: 128;
- Positional Encoding Dimension: 128;
- Context Embedding Synthesis Weight: 1;
- ELMo Layers: 2;
- Encoder Dimension: 128;
- Traditional Self-Attention Heads: 4;
- Intra-Phrase Self-Attention Heads: 4;
- Learning Rate: $1\times 10^{-5}$;
- Batch Size: 32;
- Loss Weights (α and β): Both set to 1;
- Padding Length: 300.

### 4.3. Results

We compared the rule-based approach with other machine learning approaches, and here are the performances of the various models, including precision, recall, and F1 score. The calculation methods for these metrics are defined as follows:

True Positive (TP): The number of notes correctly predicted as boundaries that are indeed actual boundaries. These metrics reflect the correctness and coverage of predicted phrase boundaries, respectively.

False Positive (FP): The number of notes predicted as boundaries but are not actual boundaries.

False Negative (FN): The number of notes that are actual boundaries but were not predicted as boundaries by the model.

(26) $$\mathrm{Precision}=\frac{TP}{TP+FP}$$

Precision measures the proportion of predicted phrase boundaries that are actual boundaries. It reflects the correctness of predicted phrase boundaries.

(27) $$\mathrm{Recall}=\frac{TP}{TP+FN}$$

Recall represents the proportion of actual phrase boundaries correctly predicted as boundaries. It reflects the coverage of true phrase boundaries.

Focusing solely on either precision or recall can lead to misleading conclusions. A model with high precision might miss many true phrase boundaries, while a model with high recall might predict numerous incorrect boundaries. The F1 score balances these two metrics, providing a more comprehensive evaluation of model performance. It combines precision and recall into their harmonic mean, making it particularly valuable when there is a trade-off between the two.

(28) $$F_{1}=\frac{2\times \mathrm{Precision}\times \mathrm{Recall}}{\mathrm{Precision}+\mathrm{Recall}}$$

The model proposed in this paper is compared with several mainstream phrase segmentation methods, including both rule-based and machine learning approaches. The comparison includes the Pause-based method, which identifies phrase boundaries based solely on the presence of long rests in the melody, and LBDM, a rule-based local boundary detection model. Notably, both Pause and LBDM do not require a train–test split or cross-validation, as they are applied directly to the entire dataset without model training.

Among the learning-based methods, CNN-CRF and BiLSTM-CRF were evaluated using a fixed train–test split without cross-validation. The Random Forest model, however, was trained using cross-validation, and its reported results represent the average performance across folds.

Thus, the results presented for our model, TransPhrase, are also averaged across 5-fold cross-validation. This setup provides a robust and comparable basis for assessing model performance. The detailed evaluation metrics, including precision, recall, and F1 score, are shown in Table 1.

The results are summarized in Table 1. In the task of music phrase segmentation, accuracy and recall are key metrics for evaluating model performance. Accuracy reflects the correctness of the predicted phrase boundaries, while recall measures the model’s ability to capture all correct boundaries. For example, the Pause model, which relies on long rests to identify phrase boundaries, achieves high accuracy (98%) because it accurately predicts boundaries based on this feature. However, since it depends solely on the local feature of long rests, its recall is lower (48%), indicating that many phrase boundaries remain unrecognized. This highlights why many handcrafted feature-based models struggle to achieve good recall performance, as they often rely on fixed feature templates that fail to comprehensively capture all potential phrase boundary indicators.

In contrast, the deep learning-based BiLSTM-CRF method leverages contextual information more effectively and can identify more phrase boundaries, resulting in improved performance. The BiLSTM-CRF model excels in capturing local context dependencies but is still limited by its inability to model global context effectively. This limitation affects both recall and accuracy.

Our model, TransPhrase (short for Transformer-based Phrase Segmentation), by simultaneously considering both local context and global structure, addresses the limitations of previous approaches. As a result, it achieves measurable improvements, with an accuracy of 87% and a recall of 86%. While the performance gains are moderate in absolute terms, they demonstrate that the integration of ELMo embeddings and intra-phrase self-attention mechanisms provides complementary benefits. More importantly, this work introduces a novel direction for applying Transformer-based architectures to symbolic music phrase segmentation, showing that a unified modeling of local and global dependencies can lead to more balanced and musically coherent boundary predictions.

### 4.4. Ablation Study

To validate the effectiveness of the components introduced in the proposed system, we conducted ablation experiments. The experiments were performed on the ESFC dataset. To ensure a fair comparison, both the baseline and our proposed model use a single-layer Transformer encoder followed by a CRF decoder. The key difference lies in the embedding and attention strategy: the baseline uses static embeddings, while our model employs dynamic ELMo embeddings and introduces intra-phrase self-attention with a guided loss. As a result, our model required 40–50 min per fold for training, slightly more than the baseline’s 35–45 min. The baseline experiment employs the standard Transformer architecture with 8 layers of multi-head self-attention. The results of the ablation experiments are shown in Table 2.

As shown in Table 2, the introduction of ELMo resulted in a slight improvement in precision, reaching 85.61%, while recall increased from 79.34% to 81.29%. This indicates that ELMo enhances the model’s ability to capture phrase boundaries, particularly in complex cases or those lacking clear segmentation features, leading to an increase in the F1 score to 83.39%.

With the addition of the intra-phrase self-attention heads, precision further improved to 85.93%, and recall significantly increased to 84.02%, yielding an F1 score of 84.96%. This demonstrates that the intra-phrase self-attention head plays a crucial role in capturing local contextual information, particularly in modeling dependencies between notes within phrases. As a result, the model is able to predict phrase boundaries more accurately and effectively handle complex phrase segmentation scenarios.

Finally, when both ELMo and the intra-phrase self-attention heads were integrated, precision reached 86.59%, recall increased to 84.49%, and the F1 score rose to 85.52%. This indicates that the combination of ELMo and intra-phrase self-attention is more effective than using either module alone. The results suggest that the two components contribute complementary strengths: ELMo provides dynamic contextualized embeddings, while the intra-phrase self-attention heads enhance the model’s ability to capture local structural dependencies. Together, they enable the model to better handle complex musical structures and achieve more stable and accurate phrase boundary recognition.

### 4.5. Attention Heatmap Visualization

**Figure 2:**
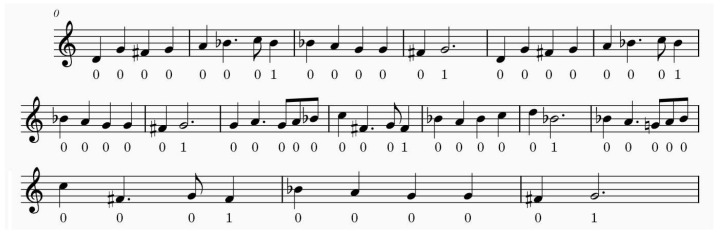
Example musical score.

In this section, we visualize the outputs of one intra-phrase self-attention head within the multi-head self-attention layer, along with the outputs of one traditional self-attention head. An example of the musical score is shown in Figure 2.

**Figure 3:**
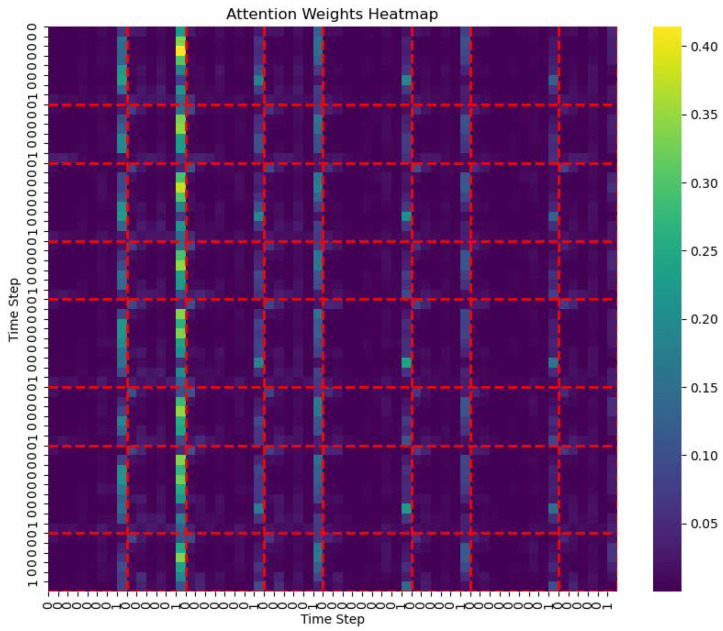
Traditional self-attention heatmap.

From the heatmap of the traditional self-attention head (Figure 3). The red dashed lines indicate the positions of notes labeled as phrase boundaries. It can be observed that the model tends to focus on global information. The heatmap shows the distribution of attention weights across time steps, revealing a relatively uniform pattern, especially over larger spans. This indicates that the traditional self-attention mechanism is inclined to capture long-range global dependencies, such as repeated melodic structures. While this mechanism handles global context well, it may overlook local contextual information, particularly in phrase structures where short-term dependencies are tightly connected.

**Figure 4:**
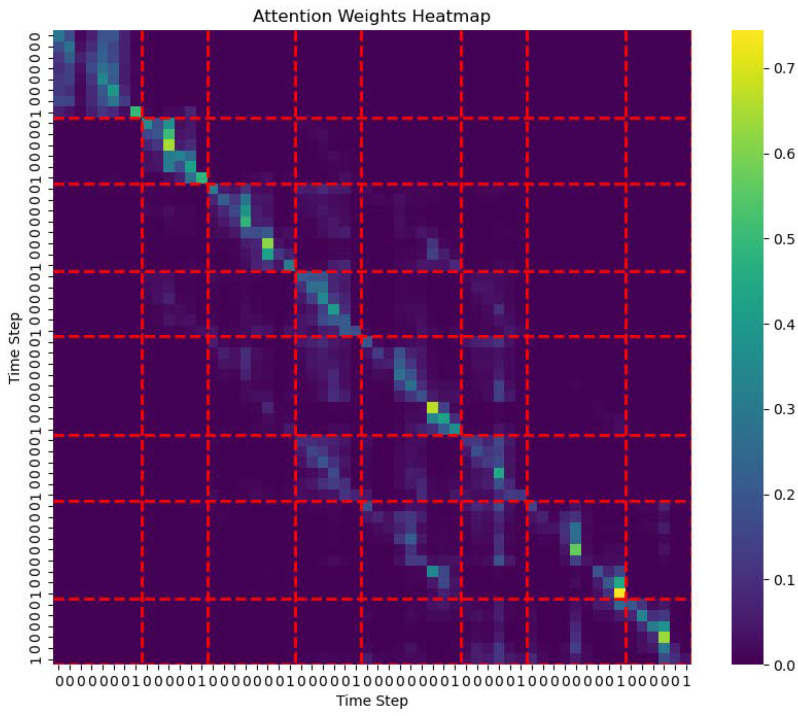
Intra-phrase self-attention heatmap.

In contrast, the heatmap of the intra-phrase self-attention head (Figure 4) focuses on a narrower time span within the phrase, exhibiting a clear block-like structure that highlights strong interrelationships between notes within the phrase. This structure underscores the model’s advantage in capturing local contextual dependencies within phrases. The intra-phrase self-attention head is able to accurately capture the relationships between notes within a phrase, reflecting the tight dependencies of the local phrase structure. The stronger attention within localized regions of the heatmap indicates that this module effectively identifies semantic connections within the phrase.

### 4.6. Discussion of Results

**Figure 5:**
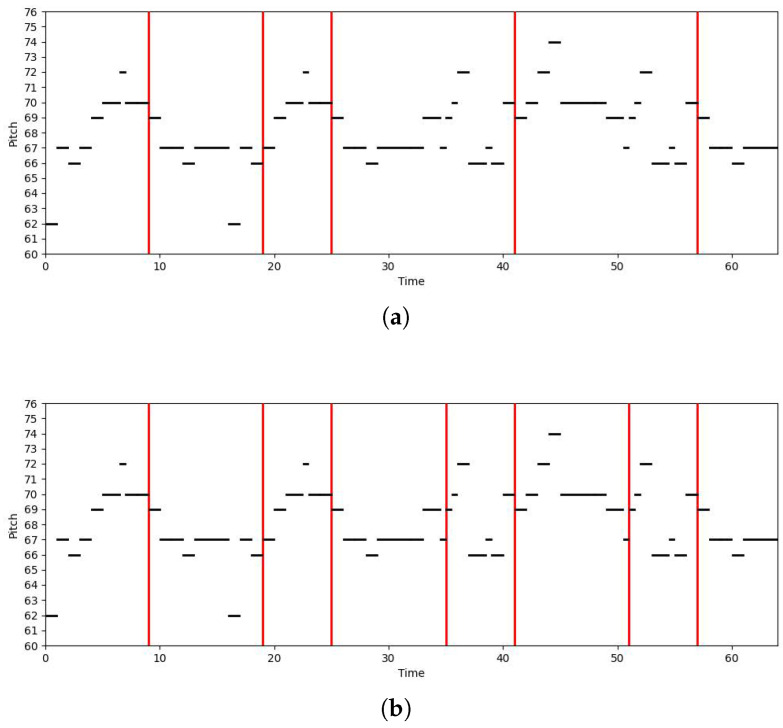
Segmentation results of different models for the same musical score: (**a**) Output of the BiLSTM-CRF model. (**b**) Output of our model and the ground truth.

To visually demonstrate the segmentation results of the model, we visualize the model’s output using a piano roll. The example musical score used is the one shown in Figure 2, and the results are presented in Figure 5.

The Figure 5 displays the output results of our model and the BiLSTM-CRF model. It is evident that the BiLSTM-CRF model misses some phrase boundaries, particularly at less obvious segmentation points. Although it performs well in capturing local context, its understanding of the global structure is limited, which leads to its failure in identifying all the phrase boundaries.

In contrast, our model detects more phrase boundaries, indicating that it is more sensitive to the phrase structure. Our model not only captures the obvious phrase breaks but also identifies the more subtle boundaries, resulting in a more detailed and accurate phrase segmentation.

## 5. Conclusions and Future Work

This paper proposes a symbolic music phrase segmentation method based on local contextual enhancement and global structural awareness. The method dynamically extracts the embedding word vectors for each note based on the contextual information of the musical score. It then uses a Transformer model, incorporating both intra-phrase self-attention heads and traditional self-attention heads, to extract both global contextual information and local phrase-level contextual information for phrase segmentation. Through comparison experiments, attention heatmap visualizations, and ablation experiments, we demonstrate that the proposed algorithm outperforms several traditional methods and surpasses the current best-performing BiLSTM-CRF model, while also producing phrase boundaries that align more closely with musical structural characteristics.

However, we note that our current experiments are conducted on a dataset consisting exclusively of folk songs. As such, the generalizability of the model to other musical forms—such as classical, popular, or jazz—remains an open question. Future work may explore whether the model performs well on these other styles, whether training with more diverse musical data can improve robustness, and how effectively the learned representations transfer across genres. Addressing these directions will help validate the broader applicability and versatility of the proposed approach.

## Figures and Tables

**Table 1:** Detailed results of testing on the EFSC dataset.

Model	P (%)	R (%)	F1 (%)
Pause [28]	98	48	60
LBDM [19]	81	60	65
Random Forest [29]	90	70	79
RBM (10-gram) [22]	83	50	60
ETPR [24]	77	81	77
CNN-CRF [9]	-	-	82
BiLSTM-CRF [9]	85	83	84
TransPhrase (ours)	87	84	86

**Table 2:** Ablation experiment results.

Model	P (%)	R (%)	F1 (%)
Baseline	85.27	79.34	82.17
With ELMo	85.61	81.29	83.39
With Intra-Phrase Self-Attention Heads	85.93	84.02	84.96
Full Model	86.59	84.49	85.52

## Data Availability

The data presented in this study are available in this article.
